# Supplementary material for: The whole-genome molecular epidemiology of sequential isolates of Acinetobacter baumannii colonizing the rectum of patients in an adult intensive care unit of a tertiary hospital
Source: Microbiol Spectr. 2023 Oct 16;11(6):e02191-23. doi: 10.1128/spectrum.02191-23 (PMC10715177; doi:10.1128/spectrum.02191-23)
Supplement: Fig. S1 — Phylogenetic tree showing the relationship between the 202 ST2 A. baumannii isolates from this study and 288 ST2 A. baumannii isolates for which a closed genome sequence is available at NCBI. [file spectrum.02191-23-s0001.pdf]

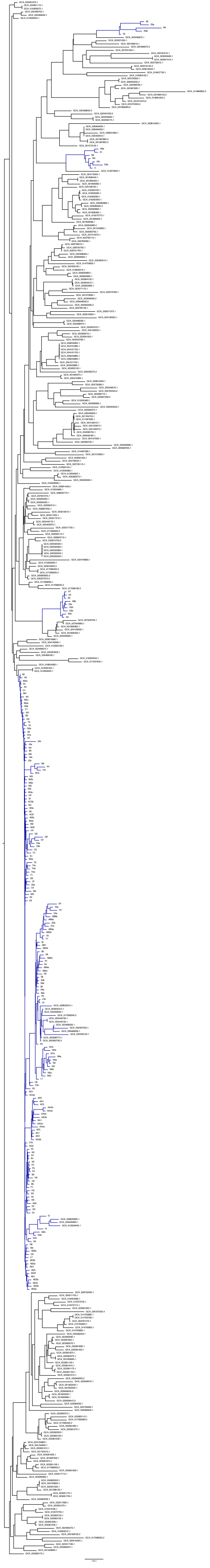

Figure S1. A phylogenetic tree showing the relationship between the 202 ST2 *A. baumannii* isolates from this study and 288 ST2 *A. baumannii* isolates for which a closed genome sequence is available at NCBI. Metadata relating to the 288 closed genome sequences is presented in Table S2. The tree was inferred using a reference-free K-mer-based genome comparison method implemented in Mashtree. The subgroup for each isolate is indicated by colored branches with Green (Main), Red (G1), Dark Blue (G2) and light blue (G3) blue. Where available, geolocation and year of isolation are shown on the taxon labels of the closed genomes.
