# Supplementary material for: The whole-genome molecular epidemiology of sequential isolates of Acinetobacter baumannii colonizing the rectum of patients in an adult intensive care unit of a tertiary hospital
Source: Microbiol Spectr. 2023 Oct 16;11(6):e02191-23. doi: 10.1128/spectrum.02191-23 (PMC10715177; doi:10.1128/spectrum.02191-23)
Supplement: Table S1 — Scheme for isolation of A. baumannii. [file spectrum.02191-23-s0002.docx]

Table S1. The scheme for isolation of *A. baumannii* from 32 patients*

| **Patient** | **†Consecutive isolations (with different colony morphology§) and STs^¥^of colonies on indicated sampling days against each patient** | | | | | | | | | | |
| --- | --- | --- | --- | --- | --- | --- | --- | --- | --- | --- | --- |
|  | **1^st^** | **2^nd^** | **3^rd^** | **4^th^** | **5^th^** | **6^th^** | **7^th^** | **8^th^** | **9^th^** | **10^th^** | **11^th^** |
| **A** | 0 day  ST113 | 7 days  ST113 | 28 days  ST2 | 32 days  ST2 | 37 days (2)  ST2&ST113 |  |  |  |  |  |  |
| **B** | 0 day  ST2 | 4 days  ST2 | 11 days  ST2 | 21 days  ST2 | 24 days  ST2 | 28 days  ST584 | 39 days  ST 2 |  |  |  |  |
| **C** | 0 day  ST2 | 22 days  ST78 | 50 days  ST2 | 74 days  ST2 | 88 days (3)  All ST2 | 109 days  ST2 |  |  |  |  |  |
| **D** | 0 day  ST2 | 7 days  ST2 | 10 days  ST2 | 15 days  ST2 | 18 days  ST2 | 22 days  ST2 | 25 days  ST2 |  |  |  |  |
| **E** | 0 day  ST2 | 7 days (2)  ST2&ST584 | 14 days  ST2 | 35 days  ST2 | 49 days  ST2 | 73 days  ST584 |  |  |  |  |  |
| **F** | 0 day  ST2 | 3 days  ST2 | 7 days  ST2 | 10 days  ST2 | 14 days  ST2 |  |  |  |  |  |  |
| **G** | 0 day (2)  Both ST2 | 3 days  ST2 | 35 days (2)  Both ST2 | 41 days  ST2 | 49 days (3)  All ST2 |  |  |  |  |  |  |
| **H** | 0 day  ST2 | 7 days  ST2 | 16 days  ST2 | 27 days  ST2 | 161  ST2 | 182  ST2336 |  |  |  |  |  |
| **I** | 0 day  ST2 | 10 days (2)  Both ST2 | 24 days (2)  Both ST2 | 115 days  ST2 | 154 days  ST2 | 189 days  ST113 | 283 days (2)  Both ST113 | 301 days  ST2 | 343 days  ST113 |  |  |
| **J** | 0 day  ST2 | 11 days  ST2 | 35 days (2)  Both ST2 | 42 days (2)  ST2 | 119 days  ST2334 | 161 days  ST2334 | 175 days  ST2334 | 210 days  ST2334 | 269 days (2)  Both ST647 | 311 days (2)  ST647 | 314 days (2)  Both ST647 |
| **K** | 0  ST2 | 20  ST2 | 42  ST2 | 66  ST2 | 122  ST2177 | 190  ST2 |  |  |  |  |  |
| **L** | 0 day  ST2 | 19 days  ST25 | 27 days  ST2 | 33 days (2)  Both ST25 | 37 days (3)  ST25, ST2&ST25 | 41 days  ST25 | 47 days  ST2 | 54 days  ST25 | 61 days  ST25 |  |  |
| **M** | 0 day  ST2 | 31 days  ST2 | 34 days (5)  All ST2 | 38 days  ST2 | 91 days  ST2 | 112 days  ST2 |  |  |  |  |  |
| **N** | 0 day  ST2 | 3 days  ST2 | 15 days  ST2 | 71 days (2)  Both ST2 | 78 days (2)  Both ST2 | 82 days (2)  Both ST2 | 89 days (2)  Both ST2 | 120 days  Both ST2 | 138 days  Both ST2 |  |  |
| **O** | 0 day  ST1093 | 10 days  ST1093 | 59 days  ST113 | 67 days  ST1093 | 70 days  ST2 | 77 days  ST2 |  |  |  |  |  |
| **P** | 0 day  ST2 | 14 days  ST2 | 105 days  ST2 | 115 days  ST2 | 119 days  ST2 | 126 days  ST2 | 154 days  ST113 | 171days  ST2 |  |  |  |
| **Q** | 0 day  ST2 | 7 days  ST2 | 14 days  ST2 | 24 days  ST2 | 28 days  ST2 |  |  |  |  |  |  |
| **R** | 0 day  ST2 | 16 days  ST113 | 23 days (2)  ST113&ST2 | 33 days  ST2 | 51 days (2)  Both ST113 | 54 days  ST113 |  |  |  |  |  |
| **S** | 0 day  ST2 | 102 days  ST2 | 126 days (2)  Both ST2 | 137 days (2)  Both ST2 | 158 days  ST2 | 179 days  ST2 |  |  |  |  |  |
| **T** | 0 day  ST2 | 3 days  ST2 | 14 days  ST2 | 133 days (3)  All ST2 | 161 days  ST2 |  |  |  |  |  |  |
| **U** | 0 day  ST2 | 7 days  ST2 | 10 days  ST2 | 18 days  ST2 | 74 days  ST2 |  |  |  |  |  |  |
| **V** | 0 day  ST2 | 4 days  ST164 | 14 days  ST164 | 18 days  ST164 | 25 days  ST164 |  |  |  |  |  |  |
| **W** | 0 day  ST2 | 24 days  ST2 | 35 days  ST2 | 42 days (2)  Both ST2 | 49 days (3)  All ST2 | 59 days  ST2 |  |  |  |  |  |
| **X** | 0 day  ST2 | 17 days  ST2 | 28 days (4)  All ST2 | 44 days  ST2 | 52 days  ST2 | 59 days (2)  Both ST2 | 73 days  ST2 |  |  |  |  |
| **Y** | 0 day  ST2 | 4 days  ST2 | 11 days  ST2 | 14 days  ST2 | 18 days (2)  Both ST2 |  |  |  |  |  |  |
| **Z** | 0 day (2)  Both ST2 | 28 days (2)  Both ST113 | 42 days (2)  Both ST2 | 53 days  ST2 | 66 days  ST113 | 70 days  ST2 | 76 days  ST113 | 81 days  ST113 | 88 days  ST113 |  |  |
| **AA** | 0 day  ST2 | 4 days  ST267 | 11 days (4)  All ST2 | 14 days (3)  All ST2 | 37 days  ST584 |  |  |  |  |  |  |
| **AB** | 0 day  ST2 | 17 days (3)  ST113, ST2&ST2 | 20 days (2)  Both ST2 | 38 days (2)  Both ST2 | 41 days (2)  Both ST113 | 52 days (2)  Both ST2 |  |  |  |  |  |
| **AC** | 0 day  ST584 | 3 days  ST584 | 7 days (2)  Both ST2 | 17 days (2)  Both ST584 | 28 days  ST2 | 31 days  ST2 |  |  |  |  |  |
| **AD** | 0 day  ST2 | 7 days  ST2 | 35 days  ST584 | 38 days  ST584 | 49 days (3)  All ST584 | 70 days  ST2 |  |  |  |  |  |
| **AE** | 0 day  ST2 | 7 days (6)  All ST2 | 14 days  ST2 | 35 days  ST2 | 49 days (2)  Both ST2 | 63 days  ST113 |  |  |  |  |  |
| **AF** | 0 day  ST2 | 7 days  ST2 | 18 days  ST2 | 28 days  ST2 | 32 days  ST2 | 47 days  ST2 |  |  |  |  |  |

*Adapted from Al-Hashem G, Rotimi VO, Albert MJ. Microb Drug Resist. 2021; 27: 64-72.

†Day of first isolation is 0 with other sampling points counted in days from this day. If more than 1 colony was tested at a sampling point, the number of colonies tested is indicated in parenthesis. No parenthesis with no number indicates a single colony has been tested. The number of colonies tested per patient corresponded to the number of different colony morphologies. Some patients were positive beyond 11 sampling occasions. From these patients, isolates from selected days were studied not to exceed 11 sampling occasions.

¥Pasteur Sequence Type §No. of colony types
