# Supplementary material for: The whole-genome molecular epidemiology of sequential isolates of Acinetobacter baumannii colonizing the rectum of patients in an adult intensive care unit of a tertiary hospital
Source: Microbiol Spectr. 2023 Oct 16;11(6):e02191-23. doi: 10.1128/spectrum.02191-23 (PMC10715177; doi:10.1128/spectrum.02191-23)
Supplement: Table S4 — Antimicrobial resistance genes. [file spectrum.02191-23-s0005.docx]

**Table S4.** Antimicrobial resistance genes found in the 269 *A. baumannii* isolates.

| **Gene** | **Abbreviation^** | **Gene product** | **Antibiotic class** | **Antibiotic subclass** |
| --- | --- | --- | --- | --- |
| aac(3)-Ia | ag1 | Aminoglycoside N-acetyltransferase AAC(3)-Ia | Aminoglycoside | Gentamycin |
| aac(3)-IIe | ag2 | Aminoglycoside N-acetyltransferase AAC(3)-IIe | Aminoglycoside | Gentamycin |
| aac(6')-Ian | ag3 | Aminoglycoside N-acetyltransferase AAC(6')-Ian | Aminoglycoside | Amikacin/kanamycin/tobramycin |
| aacA16 | ag4 | AAC(6')-Ia family aminoglycoside 6'-N-acetyltransferase AacA16 | Aminoglycoside | Aminoglycoside |
| ant(2'')-Ia | ag5 | Aminoglycoside nucleotidyltransferase ANT(2'')-Ia | Aminoglycoside | Gentamycin/kanamycin/tobramycin |
| ant(3'')-IIa | ag6 | Aminoglycoside nucleotidyltransferase ANT(3'')-IIa | Aminoglycoside | Streptomycin/pectinomycin |
| aph(3')-Ia | ag7 | Aminoglycoside O-phosphotransferase APH(3')-Ia | Aminoglycoside | Kanamycin |
| aph(3'')-Ib | ag8 | Aminoglycoside O-phosphotransferase APH(3'')-Ib | Aminoglycoside | Streptomycin |
| aph(6)-Id | ag9 | Aminoglycoside O-phosphotransferase APH(6)-Id | Aminoglycoside | Streptomycin |
| armA | ag10 | ArmA family 16S rRNA (guanine(1405)-N(7))-methyltransferase | Aminoglycoside | Gentamycin |
| arr-2 | rf1 | NAD(+)--rifampin ADP-ribosyltransferase Arr-2 | Rifamycin | Rifamycin |
| blaCARB-16 | bcar1 | RTG family carbenicillin-hydrolyzing class A beta-lactamase | B-lactam | B-lactam |
| blaTEM-12 | btm1 | TEM-1 family broad-spectrum class A beta-lactamase | B-lactam | B-lactam |
| blaTEM-110 | btm2 | TEM family broad-spectrum class A beta-lactamase | B-lactam | B-lactam |
| blaOXA-23 | bx1 | OXA-23 family carbapenem-hydrolyzing class D beta-lactamase | B-lactam | Carbapenem |
| blaOXA-106 | bx2 | OXA-51 family carbapenem-hydrolyzing class D beta-lactamase | B-lactam | Carbapenem |
| blaOXA-125 | bx3 | OXA-51 family carbapenem-hydrolyzing class D beta-lactamase | B-lactam | Carbapenem |
| blaOXA-180 | bx4 | OXA-51 family carbapenem-hydrolyzing class D beta-lactamase | B-lactam | Carbapenem |
| blaOXA-208 | bx5 | OXA-51 family carbapenem-hydrolyzing class D beta-lactamase | B-lactam | Carbapenem |
| blaOXA-51 | bx6 | OXA-51 family carbapenem-hydrolyzing class D beta-lactamase | B-lactam | Carbapenem |
| blaOXA-64 | bx7 | OXA-51 family carbapenem-hydrolyzing class D beta-lactamase | B-lactam | Carbapenem |
| blaOXA-66 | bx8 | OXA-51 family carbapenem-hydrolyzing class D beta-lactamase | B-lactam | Carbapenem |
| blaOXA-67 | bx9 | OXA-51 family carbapenem-hydrolyzing class D beta-lactamase | B-lactam | Carbapenem |
| blaOXA-69 | bx10 | OXA-51 family carbapenem-hydrolyzing class D beta-lactamase | B-lactam | Carbapenem |
| blaOXA-71 | bx11 | OXA-51 family carbapenem-hydrolyzing class D beta-lactamase | B-lactam | Carbapenem |
| blaOXA-735 | bx12 | OXA-51 family carbapenem-hydrolyzing class D beta-lactamase | B-lactam | Carbapenem |
| blaOXA-90 | bx13 | OXA-51 family carbapenem-hydrolyzing class D beta-lactamase | B-lactam | Carbapenem |
| blaOXA-91 | bx14 | OXA-51 family carbapenem-hydrolyzing class D beta-lactamase | B-lactam | Carbapenem |
| blaADC-117 | bd1 | ADC family extended-spectrum class C beta-lactamase | B-lactam | Cephalosporin |
| blaADC-122 | bd2 | ADC family extended-spectrum class C beta-lactamase | B-lactam | Cephalosporin |
| blaADC-158 | bd3 | ADC family extended-spectrum class C beta-lactamase | B-lactam | Cephalosporin |
| blaADC-169 | bd4 | ADC family extended-spectrum class C beta-lactamase | B-lactam | Cephalosporin |
| blaADC-184 | bd5 | ADC family extended-spectrum class C beta-lactamase | B-lactam | Cephalosporin |
| blaADC-199 | bd6 | ADC family extended-spectrum class C beta-lactamase | B-lactam | Cephalosporin |
| blaADC-238 | bd7 | ADC family extended-spectrum class C beta-lactamase | B-lactam | Cephalosporin |
| blaADC-247 | bd8 | ADC family extended-spectrum class C beta-lactamase | B-lactam | Cephalosporin |
| blaADC-25 | bd9 | ADC family extended-spectrum class C beta-lactamase | B-lactam | Cephalosporin |
| blaADC-26 | bd10 | ADC family extended-spectrum class C beta-lactamase | B-lactam | Cephalosporin |
| blaADC-30 | bd11 | ADC family extended-spectrum class C beta-lactamase | B-lactam | Cephalosporin |
| blaADC-52 | bd12 | ADC family extended-spectrum class C beta-lactamase | B-lactam | Cephalosporin |
| blaADC-57 | bd13 | ADC family extended-spectrum class C beta-lactamase | B-lactam | Cephalosporin |
| blaADC-73 | bd14 | ADC family extended-spectrum class C beta-lactamase | B-lactam | Cephalosporin |
| blaPER-7 | bp1 | PER family extended-spectrum class A beta-lactamase | B-lactam | Cephalosporin |
| cmlA5 | cfx | chloramphenicol efflux MFS transporter CmlA5 | Phenicol | Chloramphenicol |
| mph(E) | mcl1 | Mph(E) family macrolide 2'-phosphotransferase | Macrolide | Macrolide |
| msr(E) | mcl2 | ABC-F type ribosomal protection protein Msr(E) | Macrolide | Macrolide |
| sul1 | sul1 | Sulfonamide-resistant dihydropteroate synthase Sul1 | Sulfonamide | Sulfonamide |
| sul2 | sul2 | Sulfonamide-resistant dihydropteroate synthase Sul2 | Sulfonamide | Sulfonamide |
| tet(39) | tet1 | Tetracycline efflux MFS transporter Tet(39) | Tetracycline | Tetracycline |
| tet(B) | tet2 | Tetracycline efflux MFS transporter Tet(B) | Tetracycline | Tetracycline |

^Used in Table S6, Table S7 and Table S8
