# Supplementary material for: The whole-genome molecular epidemiology of sequential isolates of Acinetobacter baumannii colonizing the rectum of patients in an adult intensive care unit of a tertiary hospital
Source: Microbiol Spectr. 2023 Oct 16;11(6):e02191-23. doi: 10.1128/spectrum.02191-23 (PMC10715177; doi:10.1128/spectrum.02191-23)
Supplement: Table S7 — Colony morphology and genotype. [file spectrum.02191-23-s0008.docx]

Table S7. Colony morphology and genotype: Did colony morphotype difference correlate with a difference in genotype (sequence type)?

| Colony | ST | ST2 subgroup | Antimicrobial resistance (AMR) genes* (total no.) | Different colony types at the indicated sampling times in a patient have different STs & AMR genes |
| --- | --- | --- | --- | --- |
| A5a | 113 |  | ag5,ag6,ag8,ag9,bd13,bx1,bx7,sul2,tet2 (9) |  |
| A5b | 2 | Main | ag6,ag7,ag8,ag9,ag10,bd14,bx1,bx8,btm1,mcl1,mcl2,sul2,tet2 (13) | Yes |
| C5a | 2 | Main | ag6,ag7,ag8,ag9,ag10,bd14,bx1,bx8,btm1,mcl1,mcl2,tet2 (12) |  |
| C5b | 2 | Main | ag6,ag7,ag8,ag9,ag10,bd14,bx1,bx8,btm1,mcl1,mcl2,tet2 (12) |  |
| C5c | 2 | Main | ag6,ag7,ag8,ag9,ag10,bd14,bx1,bx8,btm1,mcl1,mcl2,tet2 (12) | No |
| E2a | 2 | G1 | ag1,ag3,ag6,ag8,ag9,bd11,bx1,bx8,tet2 (9) |  |
| E2b | 584 |  | ag6,bd4,bx3 (3) | Yes |
| G1a | 2 | Main | ag6,ag7,ag8,ag9,ag10,bd14,bx1,bx8,btm1,mcl1,mcl2,tet2 (12) |  |
| G1b | 2 | Main | ag6,ag7,ag8,ag9,ag10,bd14,bx1,bx8,btm1,mcl1,mcl2,tet2 (12) | No |
| G3a | 2 | Main | ag6,ag7,ag8,ag9,ag10,bd14,bx1,bx8,btm1,mcl1,mcl2,tet2 (12) |  |
| G3b | 2 | Main | ag6,ag7,ag8,ag9,ag10,bd14,bx1,bx8,btm1,mcl1,mcl2,tet2 (12) | No |
| G5a | 2 | Main | ag6,ag7,ag8,ag9,ag10,bd14,bx1,bx8,btm1,mcl1,mcl2,tet2 (12) |  |
| G5b | 2 | Main | ag6,ag7,ag8,ag9,ag10,bd14,bx1,bx8,btm1,mcl1,mcl2,tet2 (12) |  |
| G5c | 2 | Main | ag6,ag7,ag8,ag9,ag10,bd14,bx1,bx8,btm1,mcl1,mcl2,tet2 (12) | No |
| I2a | 2337 |  | ag6,bd13,bx9 (3) |  |
| I2b | 2 | G1 | ag1,ag3,ag6,ag8,ag9,bd11,bx1,bx8,tet2 (9) | Yes |
| I3a | 2 | G1 | ag1,ag3,ag6,ag8,ag9,bd11,bx1,bx8,tet2 (9) |  |
| I3b | 2 | Main | ag6,ag8,ag9,ag10,bd14,bx1,bx8,mcl1,mcl2,tet2 (10) | Yes |
| I7a | 113 |  | ag5,ag6,ag8,ag9,bd13,bx1,bx7,sul2,tet2 (9) |  |
| I7b | 113 |  | ag5,ag6,ag8,ag9,bd13,bx1,bx7,sul2,tet2 (9) | No |
| J3a | 2 | Main | ag6,ag7,ag8,ag9,ag10,bd14,bx1,bx8,btm1,mcl1,mcl2,tet2 (12) |  |
| J3b | 2 | Main | ag6,ag7,ag8,ag9,ag10,bd14,bx1,bx8,btm1,mcl1,mcl2,tet2 (12) | No |
| J4a | 2 | Main | ag6,ag7,ag8,ag9,ag10,bd14,bx1,bx8,btm1,mcl1,mcl2,tet2 (12) |  |
| J4b | 2 | Main | ag6,ag7,ag8,ag9,ag10,bd14,bx1,bx8,btm1,mcl1,mcl2,tet2 (12) | No |
| J9a | 647 |  | ag6,bd10,bx6,tet1 (4) |  |
| J9b | 647 |  | ag6,bd10,bx6,tet1 (4) | No |
| J10a | 647 |  | ag6,bd10,bx6,tet1 (4) |  |
| J10b | 647 |  | ag6,bd10,bx6,tet1 (4) | No |
| J11a | 647 |  | ag6,bd10,bx6,tet1 (4) |  |
| J11b | 647 |  | ag6,bd10,bx6,tet1 (4) | No |
| L4a | 25 |  | ag2,ag3,ag6,ag8,ag9,bd10,bx1,bx7,sul2,tet2 (10) |  |
| L4b | 25 |  | ag2,ag3,ag6,ag8,ag9,ag10,rf1,bd10,bp1,bx1,bx7,cfx,mcl1,mcl2,sul1,sul2,tet2 (17) | Yes |
| L5a | 25 |  | ag2,ag3,ag6,ag8,ag9,ag10,rf1,bd10,bp1,bx1,bx7,cfx,mcl1,mcl2,sul1,sul2,tet2 (17) |  |
| L5b | 2 | Main | ag6,ag7,ag8,ag9,ag10,bd14,bx1,bx8,btm1,mcl1,mcl2,sul2,tet2 (13) |  |
| L5c | 25 |  | ag2,ag3,ag6,ag8,ag9,bd10,bx1,bx7,sul2,tet2 (10) | Yes |
| M3a | 2 | Main | ag6,ag7,ag8,ag9,ag10,bd14,bx1,bx8,btm1,mcl1,mcl2,tet2 (12) |  |
| M3b | 2 | Main | ag6,ag7,ag8,ag9,ag10,bd14,bx1,bx8,btm1,mcl1,mcl2,tet2 (12) |  |
| M3c | 2 | Main | ag6,ag7,ag8,ag9,ag10,bd14,bx1,bx8,btm1,mcl1,mcl2,tet2 (12) |  |
| M3d | 2 | Main | ag6,ag7,ag8,ag9,ag10,bd14,bx1,bx8,btm1,mcl1,mcl2,tet2 (12) |  |
| M3e | 2 | Main | ag6,ag7,ag8,ag9,ag10,bd14,bx1,bx8,btm1,mcl1,mcl2,tet2 (12) | No |
| N4a | 2 | Main | ag6,ag7,ag8,ag9,ag10,bd14,bx1,bx8,btm1,mcl1,mcl2,sul2,tet2 (13) |  |
| N4b | 2 | Main | ag6,ag7,ag8,ag9,ag10,bd14,bx1,bx8,btm1,mcl1,mcl2,sul2,tet2 (13) | No |
| N5a | 2 | Main | ag6,ag7,ag8,ag9,ag10,bd14,bx1,bx8,btm1,mcl1,mcl2,sul2,tet2 (13) |  |
| N5b | 2 | Main | ag6,ag7,ag8,ag9,ag10,bd14,bx1,bx8,btm1,mcl1,mcl2,sul2,tet2 (13) | No |
| N6a | 2 | Main | ag6,ag7,ag8,ag9,bd14,bx1,bx8,btm1,sul2,tet2 (10) |  |
| N6b | 2 | Main | ag6,ag7,ag8,ag9,bd14,bx1,bx8,btm1,sul2,tet2 (10) | No |
| N7a | 2 | Main | ag6,ag7,ag8,ag9,ag10,bd14,bx1,bx8,btm1,mcl1,mcl2,sul2,tet (13) |  |
| N7b | 2 | Main | ag6,ag7,ag8,ag9,ag10,bd14,bx1,bx8,btm1,mcl1,mcl2,sul2,tet2 (13) | No |
| R3a | 113 |  | ag6,ag8,ag9,bd13,bx1,bx7,sul2,tet2 (8) |  |
| R3b | 2 | G3 | ag1,ag8,ag9,ag6,bd9,bx1,bx8,sul2 (8) | Yes |
| R5a | 113 |  | ag6,ag8,ag9,bd13,bx1,bx7,sul2,tet2 (8) |  |
| R5b | 113 |  | ag6,ag8,ag9,bd13,bx1,bx7,sul2,tet2 (8) | No |
| S3a | 2 | Main | ag6,ag7,ag8,ag9,ag10,bd14,bx1,bx8,btm1,mcl1,mcl2,tet2 (12) |  |
| S3b | 2 | Main | ag6,ag7,ag8,ag9,ag10,bd14,bx1,bx8,btm1,mcl1,mcl2,tet2 (12) | No |
| S4a | 2 | Main | ag6,ag7,ag8,ag9,ag10,bd14,bx1,bx8,btm1,mcl1,mcl2,tet2 (12) |  |
| S4b | 2 | Main | ag6,ag7,ag8,ag9,ag10,bd14,bx1,bx8,btm1,mcl1,mcl2,tet2 (12) | No |
| T4a | 2 | Main | ag6,ag8,ag9,ag10,bd14,bx1,bx8,btm1,mcl1,mcl2,tet2 (11) |  |
| T4b | 2 | Main | ag6,ag8,ag9,ag10,bd14,bx1,bx8,btm1,mcl1,mcl2,tet2 (11) |  |
| T4c | 2 | Main | ag6,ag8,ag9,ag10,bd14,bx1,bx8,btm1,mcl1,mcl2,tet2 (11) | No |
| W4a | 2 | Main | ag6,ag8,ag9,ag10,bd14,bx1,bx8,btm1,mcl1,mcl2,tet2 (11) |  |
| W4b | 2 | Main | ag6,ag8,ag9,ag10,bd14,bx1,bx8,btm1,mcl1,mcl2,tet2 (11) | No |
| W5a | 2 | Main | ag6,ag8,ag9,ag10,bd14,bx1,bx8,btm1,mcl1,mcl2,tet2 (11) |  |
| W5b | 2 | Main | ag6,ag8,ag9,ag10,bd14,bx1,bx8,btm1,mcl1,mcl2,tet2 (11) |  |
| W5c | 2 | Main | ag6,ag8,ag9,ag10,bd14,bx1,bx8,btm1,mcl1,mcl2,tet2 (11) | No |
| X3a | 2 | G2 | ag6,ag8,ag9,ag10,bd14,bx1,bx8,btm1,mcl1,mcl2,sul2,tet2 (12) |  |
| X3b | 2 | G2 | ag6,ag8,ag9,ag10,bd14,bx1,bx8,btm1,mcl1,mcl2,sul2,tet2 (12) |  |
| X3c | 2 | G2 | ag6,ag8,ag9,ag10,bd14,bx1,bx8,btm1,mcl1,mcl2,sul2,tet2 (12) |  |
| X3d | 2 | G2 | ag6,ag8,ag9,ag10,bd14,bx1,bx8,btm1,mcl1,mcl2,sul2,tet2 (12) | No |
| X6a | 2 | G1 | ag3,ag6,ag8,ag9,bd11,bx1,bx8,sul2,tet2 (9) |  |
| X6b | 2 | G2 | ag6,ag8,ag9,ag10,bd14,bx1,bx8,btm1,mcl1,mcl2,sul2,tet2 (12) | Yes |
| Y5a | 2 | Main | ag6,ag8,ag9,ag10,bd14,bx1,bx8,btm1,mcl1,mcl2,tet (11) |  |
| Y5b | 2 | Main | ag6,ag8,ag9,ag10,bd14,bx1,bx8,btm1,mcl1,mcl2,tet2 (11) | No |
| Z1a | 2 | Main | ag6,ag8,ag9,ag10,bd14,bx1,bx8,btm1,mcl1,mcl2,tet2 (11) |  |
| Z1b | 2 | Main | ag6,ag8,ag9,ag10,bd14,bx1,bx8,btm1,mcl1,mcl2,tet2 (11) | No |
| Z2a | 113 |  | ag6,ag8,ag9,bd13,bx1,bx7,sul2,tet2 (8) |  |
| Z2b | 113 |  | ag6,ag8,ag9,bd13,bx1,bx7,sul2,tet2 (8) | No |
| Z3a | 2 | G3 | ag1,ag6,ag8,ag9,bd9,bx1,bx8,sul2 (8) |  |
| Z3b | 2 | Main | ag6,ag7,ag8,ag9,ag10,bd14,bx1,bx8,btm1,mcl1,mcl2,tet2 (12) | Yes |
| AA3a | 2 | Main | ag6,ag7,ag8,ag9,ag10,bd14,bx1,bx8,btm1,mcl1,mcl2,tet2 (12) |  |
| AA3b | 2 | Main | ag6,ag7,ag8,ag9,ag10,bd14,bx1,bx8,btm1,mcl1,mcl2,tet2 (12) |  |
| AA3c | 2 | Main | ag6,ag7,ag8,ag9,ag10,bd14,bx1,bx8,btm1,mcl1,mcl2,tet2 (12) |  |
| AA3d | 2 | Main | ag6,ag7,ag8,ag9,ag10,bd14,bx1,bx8,btm1,mcl1,mcl2,tet2 (12) | No |
| AA4a | 2 | Main | ag6,ag7,ag8,ag9,ag10,bd14,bx1,bx8,btm1,mcl1,mcl2,tet2 (12) |  |
| AA4b | 2 | Main | ag6,ag7,ag8,ag9,ag10,bd14,bx1,bx8,btm1,mcl1,mcl2,tet2 (12) |  |
| AA4c | 2 | Main | ag6,ag7,ag8,ag9,bd14,bx1,bx8,btm1,tet2 (9) | Yes (1 of 3 isolates different) |
| AB2a | 113 |  | ag8,ag9,ag6,bd13,bx1,bx7,sul2,tet2 (8) |  |
| AB2b | 2 | Main | ag6,ag7,ag8,ag9,ag10,bd14,bx1,bx8,btm1,mcl1,mcl2,tet2 (12) |  |
| AB2c | 2 | Main | ag6,ag7,ag8,ag9,ag10,bd14,bx1,bx8,btm1,mcl1,mcl2,tet2 (12) | Yes (1 of 3 isolates different) |
| AB3a | 2 | Main | ag6,ag7,ag8,ag9,ag10,bd14,bx1,bx8,btm1,mcl1,mcl2,tet2 (12) |  |
| AB3b | 2 | Main | ag6,ag7,ag8,ag9,ag10,bd14,bx1,bx8,btm1,mcl1,mcl2,tet2 (12) | No |
| AB4a | 2 | Main | ag6,ag7,ag8,ag9,bd14,bx1,bx8,btm1,mcl1,mcl2,tet2 (11) |  |
| AB4b | 2 | Main | ag6,ag7,ag8,ag9,ag10,bd14,bx1,bx8,btm1,mcl1,mcl2,tet2 (12) | Yes |
| AB5a | 113 |  | ag6,ag8,ag9,bd13,bx1,bx7,sul2,tet2 (8) |  |
| AB5b | 113 |  | ag6,ag8,ag9,bd13,bx1,bx7,sul2,tet2 (8) | No |
| AB6a | 2 | Main | ag6,ag7,ag8,ag9,ag10,bd14,bx1,bx8,btm1,mcl1,mcl2,tet2 (12) |  |
| AB6b | 2 | Main | ag6,ag7,ag8,ag9,ag10,bd14,bx1,bx8,btm1,mcl1,mcl2,tet2 (12) | No |
| AC3a | 2 | Main | ag6,ag7,ag8,ag9,ag10,bd14,bx1,bx8,btm1,mcl1,mcl2,tet2 (12) |  |
| AC3b | 2 | Main | ag6,ag7,ag8,ag9,ag10,bd14,bx1,bx8,btm1,mcl1,mcl2,tet2 (12) | No |
| AC4a | 584 |  | ag6,bd4,bx3 (3) |  |
| AC4b | 584 |  | ag6,bd4,bx3 (3) | No |
| AD5a | 584 |  | ag6,bd4,bx3 (3) |  |
| AD5b | 584 |  | ag6,bd4,bx3 (3) |  |
| AD5c | 584 |  | ag6,bd4,bx3 (3) | No |
| AE2a | 2 | Main | ag6,ag8,ag9,ag10,bd14,bx1,bx8,mcl1,mcl2,tet2 (10) |  |
| AE2b | 2 | Main | ag6,ag8,ag9,ag10,bd14,bx1,bx8,mcl1,mcl2,tet2 (10) |  |
| AE2c | 2 | Main | ag6,ag8,ag9,ag10,bd14,bx1,bx8,mcl1,mcl2,tet2 (10) |  |
| AE2d | 2 | Main | ag6,ag8,ag9,ag10,bd14,bx1,bx8,mcl1,mcl2,tet2 (10) |  |
| AE2e | 2 | Main | ag6,ag8,ag9,ag10,bd14,bx1,bx8,mcl1,mcl2,tet2 (10) |  |
| AE2f | 2 | Main | ag6,ag8,ag9,ag10,bd14,bx1,bx8,mcl1,mcl2,tet2 (10) | No |
| AE5a | 2 | Main | ag6,ag8,ag9,ag10,bd14,bx1,bx8,mcl1,mcl2,tet2 (10) |  |
| AE5b | 2 | Main | ag6,ag8,ag9,ag10,bd14,bx1,bx8,mcl1,mcl2,tet2 (10) | No |

*Nomenclature as in Table S4
